# Supplementary material for: Interface engineering enabling thin lithium metal electrodes down to 0.78 μm for garnet-type solid-state batteries
Source: Nat Commun. 2024 Nov 15;15:9920. doi: 10.1038/s41467-024-54234-w (PMC11568204; doi:10.1038/s41467-024-54234-w)
Supplement: Supplementary file 1 — Supplementary Information [file 41467_2024_54234_MOESM1_ESM.pdf]

Supplementary Materials

Interface engineering enabling thin lithium metal electrodes down to 0.78 μm for garnet-type solid-state batteries

Weijie Ji,[1] Bi Luo,[1] Qi Wang,[1] Guihui Yu,[1] Zixun Zhang,[1] Yi Tian,[1] Zaowen Zhao,[2] Ruirui Zhao,[3] Shubin Wang,[4] Xiaowei Wang,[1,*] Bao Zhang,[1] Jiafeng Zhang,[1,*] Zhiyuan Sang,[5] and Ji Liang[6*]

[1]*National Engineering Laboratory for High-Efficiency Recovery of Refractory Nonferrous Metals, School of Metallurgy and Environment, Central South University, Changsha 410083, China*

[2]*Special Glass Key Lab of Hainan Province, School of Materials Science and Engineering, Hainan University, Haikou 570228, China*

[3]*School of Chemistry, Engineering Research Center of MTEES (Ministry of Education), South China Normal University, Guangzhou, Guangdong 510006, China*

[4]*State Environmental Protection Key Laboratory of Urban Ecological Environment Simulation and Protection, South China Institute of Environmental Sciences, Ministry of Ecology and Environment (MEE), Guangzhou 510655, China*

[5]*School of Materials Science & Engineering, Peking University, Beijing, 100871, China*

[6]*Key Laboratory for Advanced Ceramics and Machining Technology of Ministry of Education, School of Materials Science and Engineering, Tianjin University, Tianjin 300350, China*

**Correspondence: yjywxw@csu.edu.cn; yjyzjf@csu.edu.cn; liangji@tju.edu.cn*

Contents

I. Supplementary Figures

II. Supplementary Notes

III. Supplementary Tables

References

I. Supplementary Figures

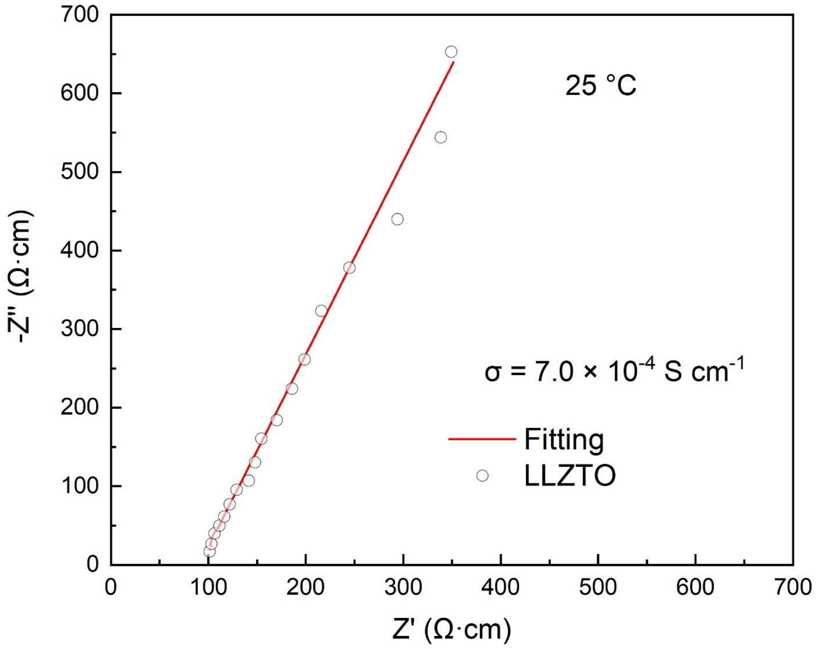

Figure S1. Electrochemical impedance of Ag|LLZTO|Ag cell at 25 °C to estimate the ionic conductivity of LLZTO.

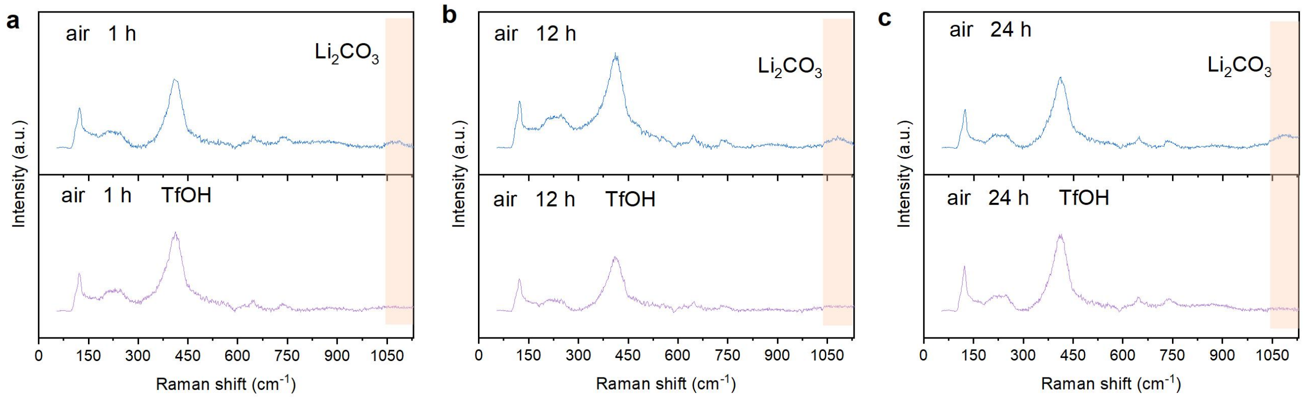

Figure S2. The Raman spectra of LLZTO after exposure for different durations. (a) Raman spectra of LLZTO exposed to air for 1 h (as shown in the figure above) and followed by TfOH surface treatment (as shown in the figure below). (b) Raman spectra of LLZTO were exposed to air for 12 h (as shown in the figure above) and followed by TfOH surface treatment (as shown in the figure below). (c) Raman spectra of LLZTO exposed to air for 24 h (as shown in the figure above) and followed by TfOH surface treatment (as shown in the figure below).

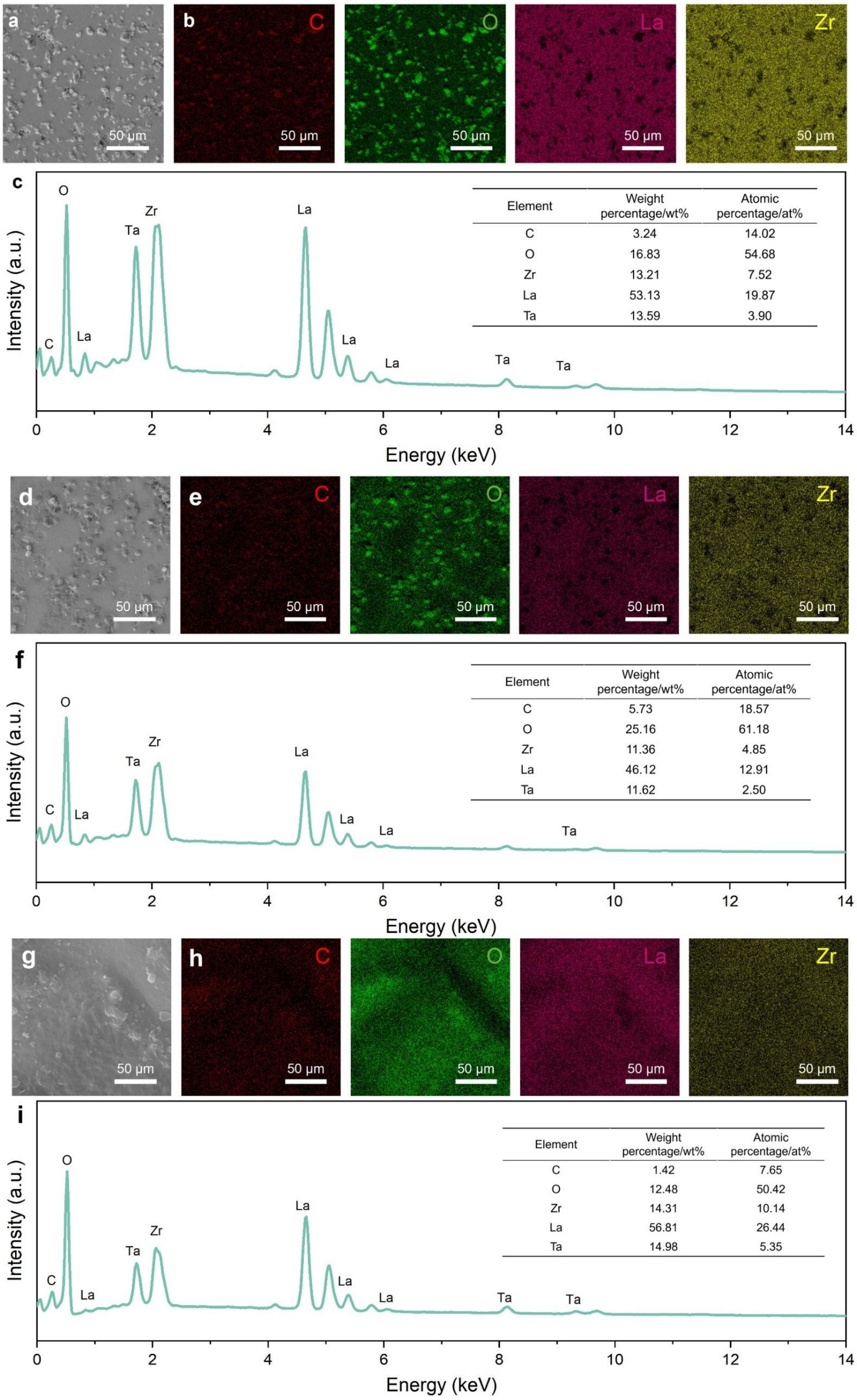

Figure S3. The SEM and EDS images of LLZTO after exposure for different durations. (a) Top-SEM, (b) EDS of elemental C, O, La, and Zr, and (c) EDS spectra of LLZTO exposed to air for 1 h. (d) Top-SEM, (e) EDS of elemental C, O, La, and Zr, and (f) EDS spectra of LLZTO exposed to air for 12 h. (g) Top-SEM, (h) EDS of elemental C, O, La, and Zr, and (i) EDS spectra of LLZTO exposed to air for 24 h.

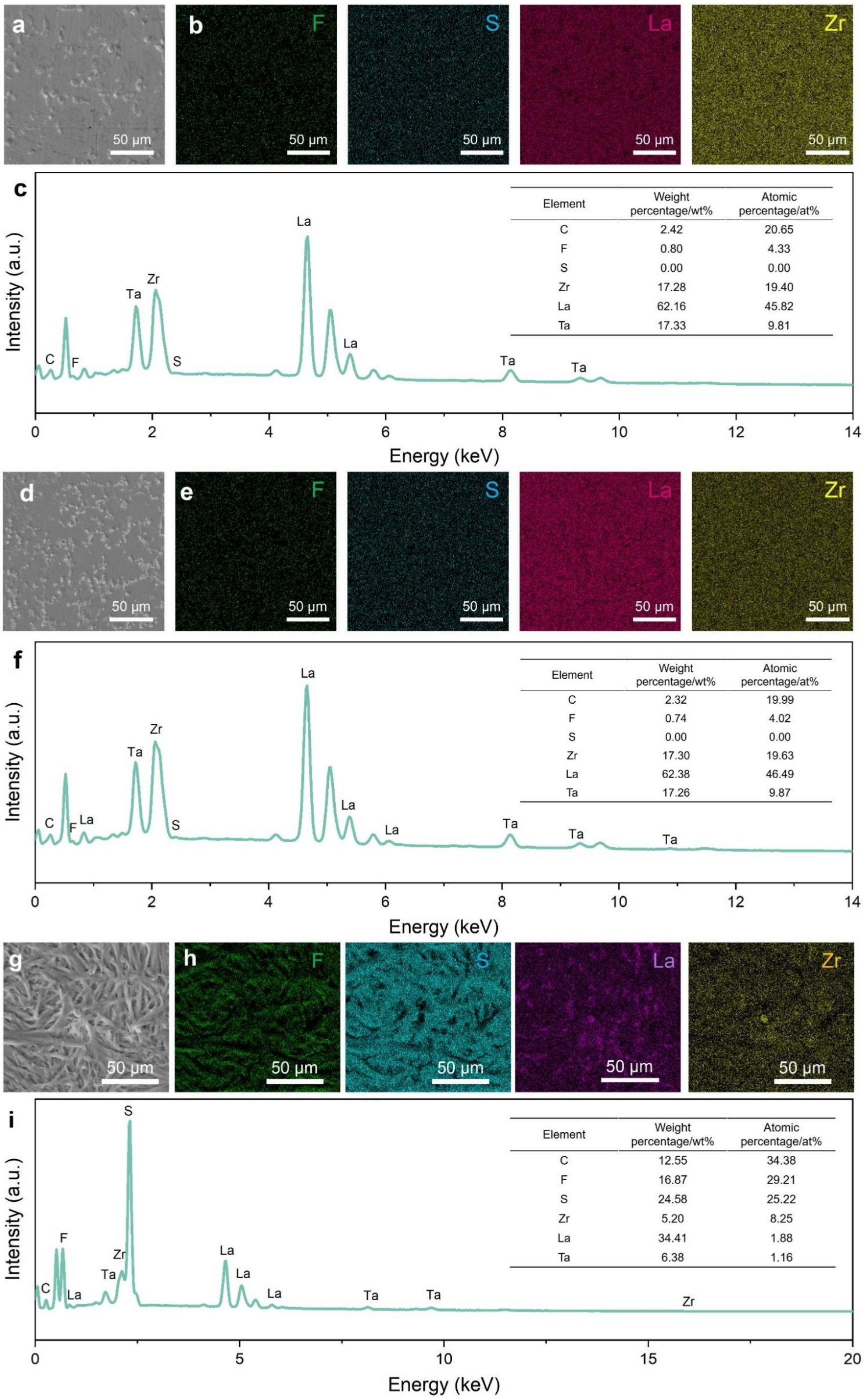

Figure S4. The SEM and EDS images of LLZTO after exposure for different durations, followed by TfOH treatment. (a) Top-SEM, (b) EDS of elemental F, S, La, and Zr, and (c) EDS spectra of LLZTO exposed to air for 1 h followed by TfOH surface treatment. (d) Top-SEM, (e) EDS of elemental F, S, La, and Zr, and (f) EDS spectra of LLZTO exposed to air for 12 h followed by TfOH surface treatment. (g) Top-SEM, (h) EDS of elemental F, S, La, and Zr, and (i) EDS spectra of LLZTO exposed to air for 24 h followed by TfOH surface treatment.

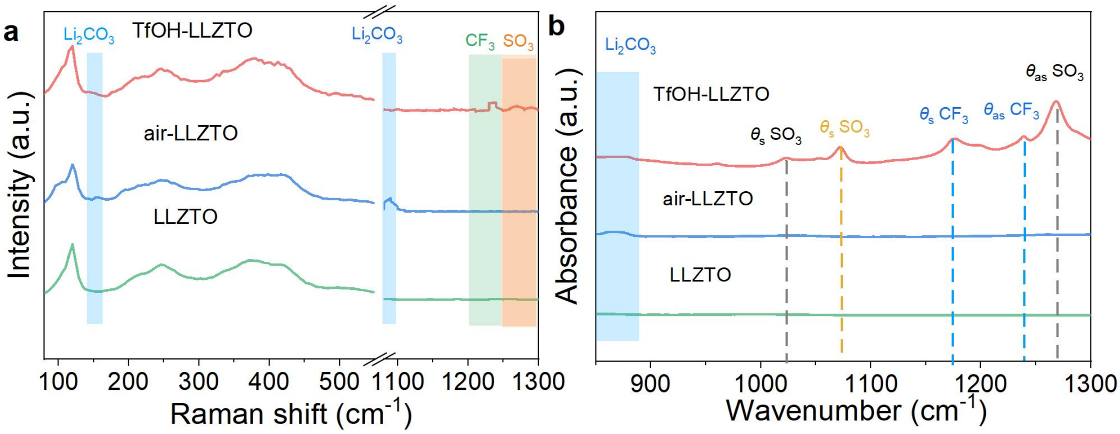

Figure S5. (a) Raman spectra and (b) Attenuated total reflectance Fourier-transform infrared spectra (ATR-FTIR) of pristine LLZTO, LLZTO-air, and TfOH-LLZTO.

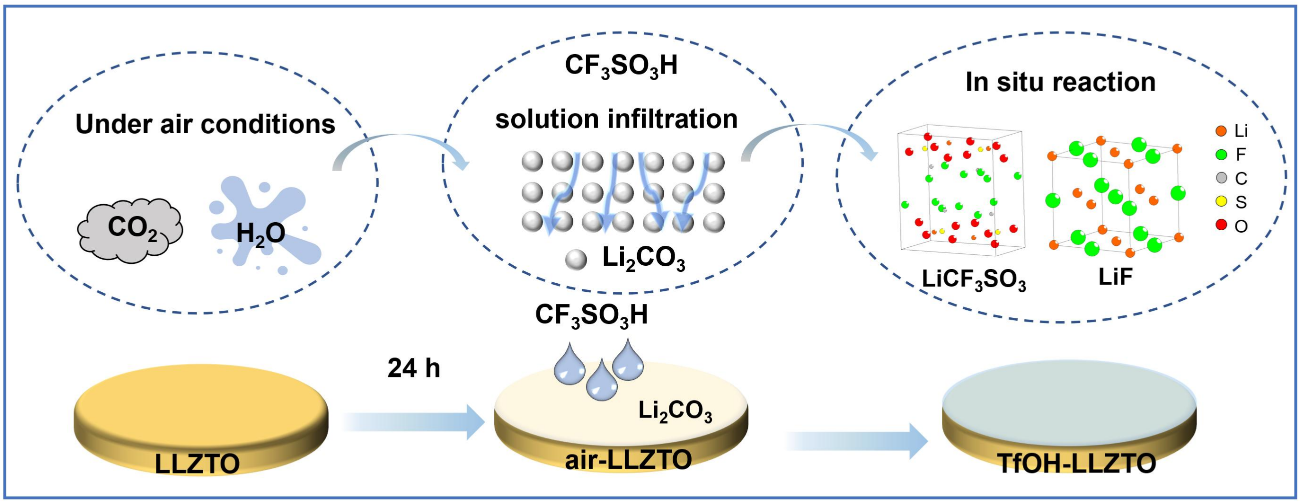

Figure S6. The schematic diagram shows the in situ conversion strategy of transforming the Li_2CO_3 passivation layer on LLZTO into a lithium friendly interlayer through Triflic acid in DMSO solution, and transforming it into a mixed ion/electronic interlayer after lithium melting at high temperature (300 °C).

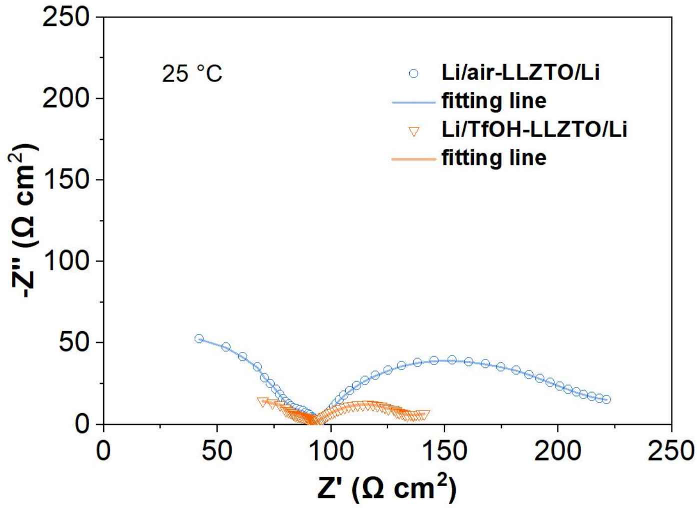

Figure S7. Nyquist plots of Li|air-LLZTO|Li and Li|TfOH-LLZTO|Li.

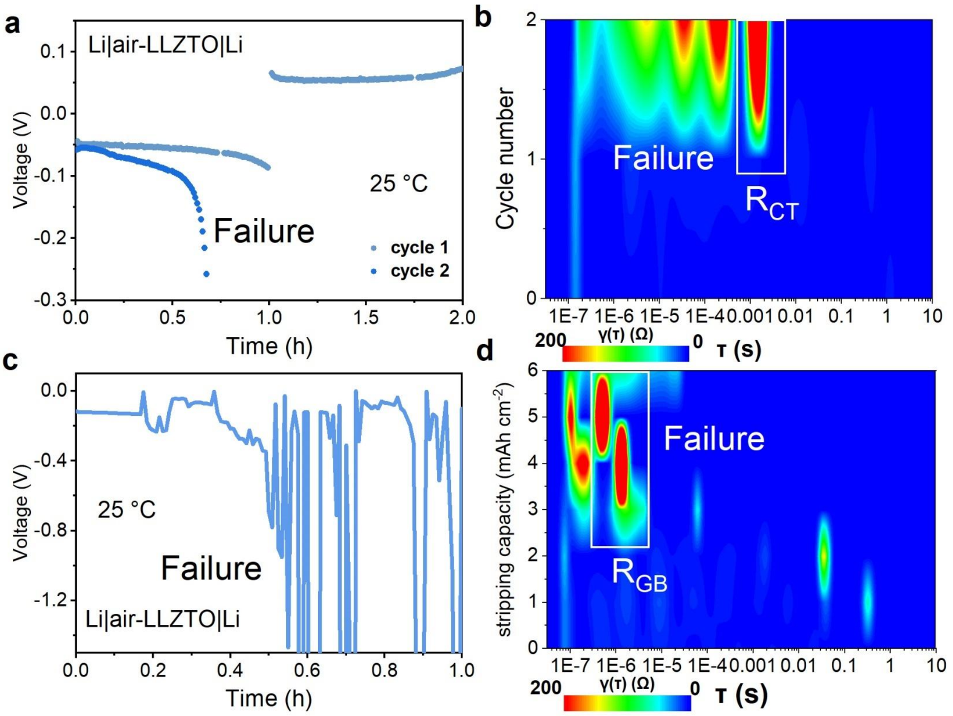

Figure S8. Electrochemical performance of Li|air-LLZTO|Li. (a) Voltage profile during dissolution and deposition cycling experiments on Li|air-LLZTO|Li cell at 0.3 mA cm^{-2}. (b) The DRT transition result of EIS was recorded in (a) at the current density of 0.3 mA cm^{-2}. In this figure, three sets of data are recorded before the cycle, after the first cycle, and after the second cycle. (c) Evolution of polarization voltage and (d) DRT transition result of EIS recorded during unidirectional charging in Li|air-LLZTO|Li cell at 0.5 mA cm^{-2}. In Figure S8d, seven sets of data are recorded by the step of stripping 1 mAh cm^{-2}.

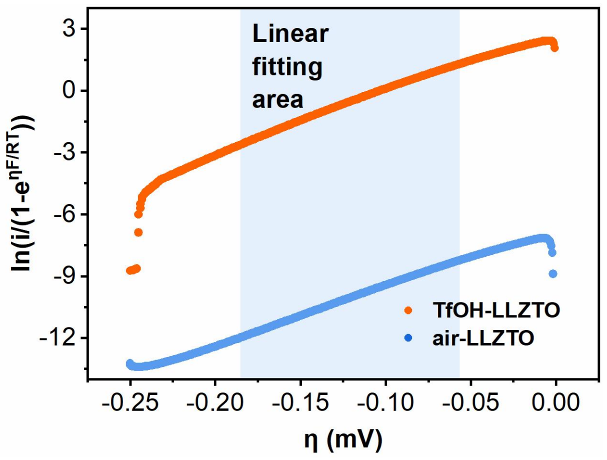

Figure S9. Corresponding AllenHickling plots with intercepts on vertical axis based on extrapolated linear fitting to determine exchange current. η is polarization.

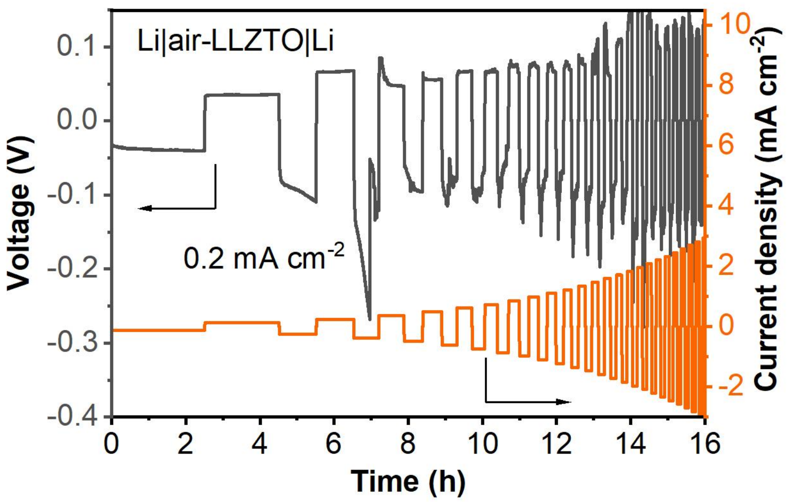

Figure S10. Voltage-time profiles of Li|air-LLZTO|Li on galvanostatic cycling with stepped current density and constant capacity at 25 °C.

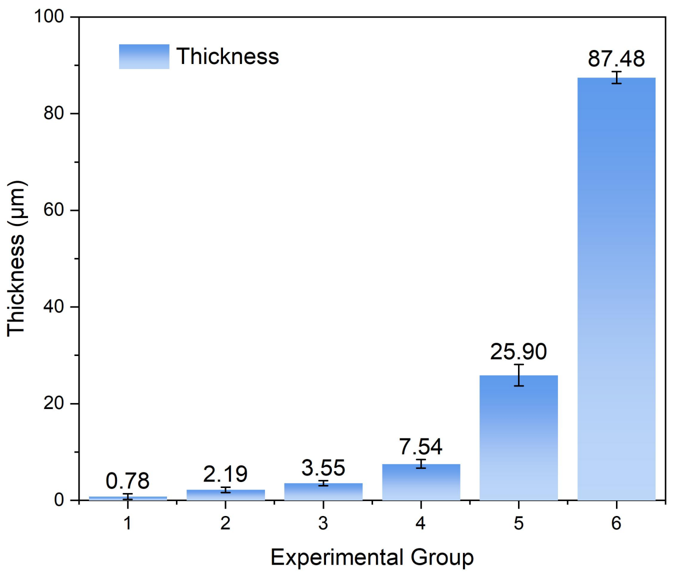

Figure S11. The thickness of the lithium metal negative electrodes in Figures 4b-g. Error bars are s.d.; n = 5.

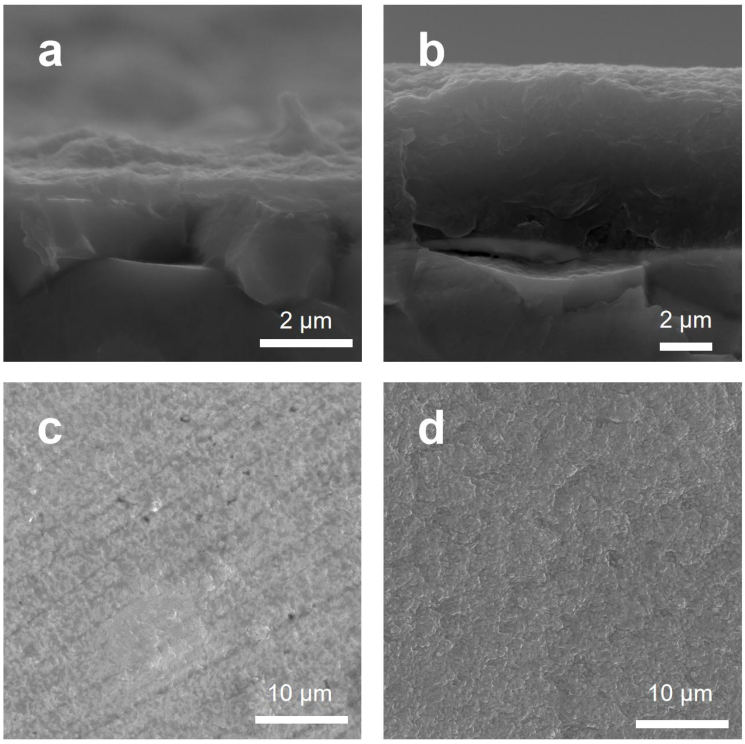

Figure S12. Cross-view SEM images of interfaces composed of LLZTO and lithium metals of (a) 0.78 μm and (b) 7.54 μm. Cross-view SEM images of interfaces composed of LLZTO and lithium metals of (c) 0.78 μm and (d) 7.54 μm.

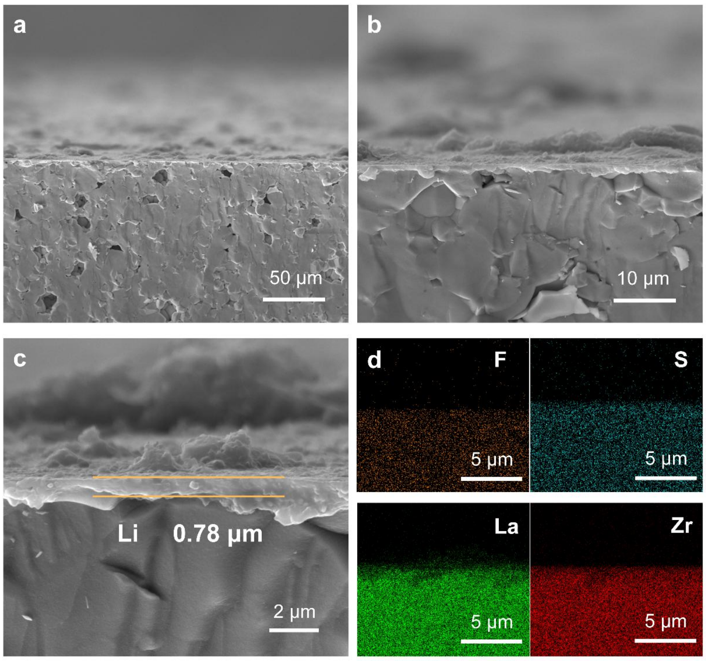

Figure S13. (a-c) Cross-sectional SEM and (d) EDS images of Li|TfOH-LLZTO interface.

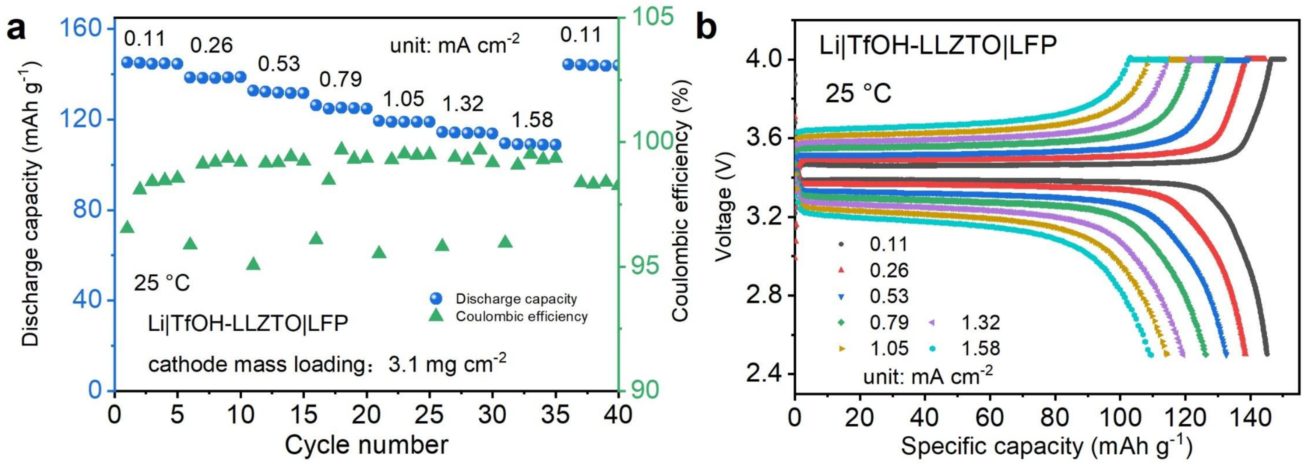

Figure S14. Electrochemical performance of Li|TfOH-LLZTO|LFP. (a) Galvanostatic cycling performance and (b) voltage profiles of Li|TfOH-LLZTO|LFP cell using unlimited Li metal (87.48 μm) at various current densities.

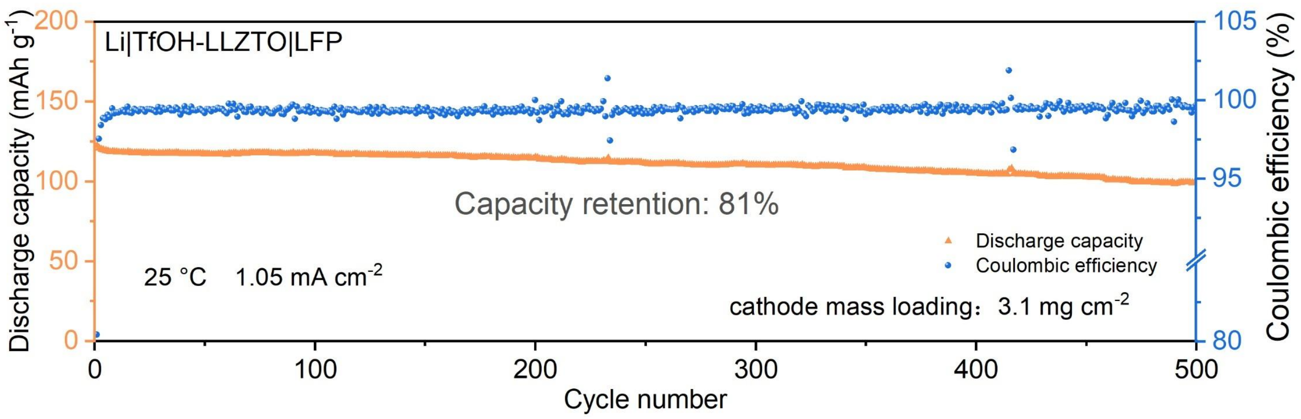

Figure S15. Cycling performances of Li|TfOH-LLZTO|LFP cells using unlimited Li metal (87.48 μm).

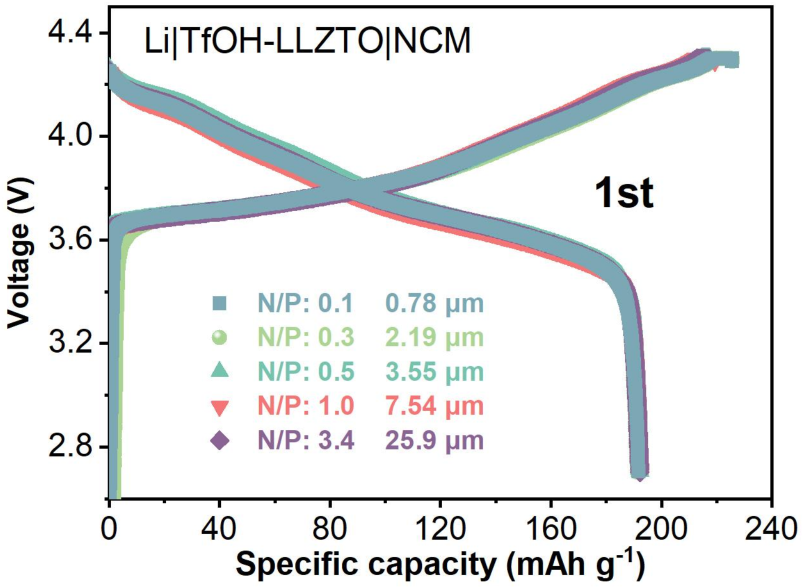

Figure S16. The first voltage profiles of Li|TfOH-LLZTO|NCM cells using limited Li metal at various N/P ratios.

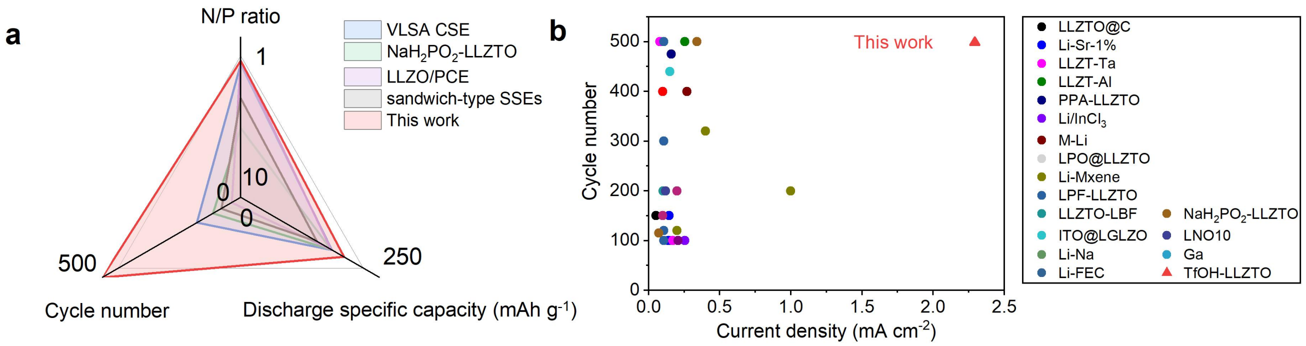

Figure S17. Comparison of the electrochemical performance of Li|TfOH-LLZTO|NCM (N/P ratio: 1.1) with other literature. (a) Comparison of cycling performance with previously reported results of state-solid electrolytes at various N/P ratios[1, 2, 3, 4]. (b) Comparison of cycling performance with previously reported results of Garnet-type state-solid electrolytes. The references for the reports are shown in Table S2.[5, 6, 7, 8, 9, 10, 11, 12, 13, 14, 15, 16, 17, 18, 19, 20, 21]

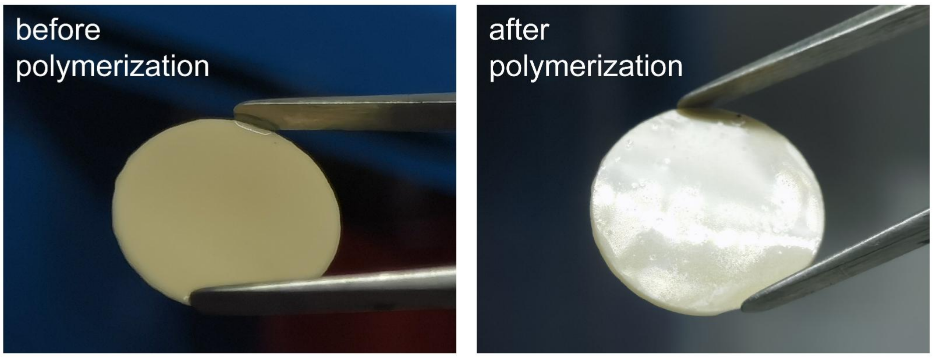

Figure S18. Optical photos of the PSE on the surface of LLZTO before and after polymerization.

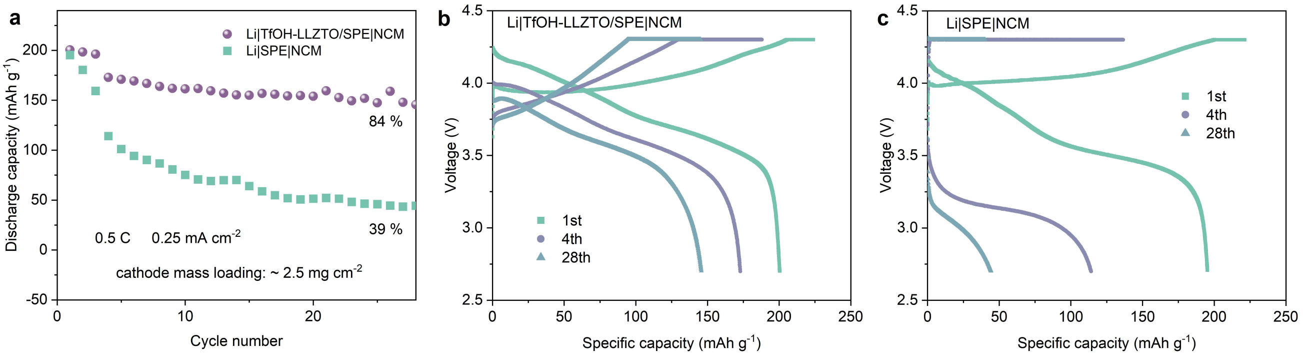

Figure S19. Cycling performances of Li|TfOH-LLZTO/SPE|NCM and Li|SPE|NCM. (a) Cycling performances of Li|TfOH-LLZTO/SPE|NCM and Li|SPE|NCM cells at 50 °C. Voltage profiles of (b) Li|TfOH-LLZTO/SPE|NCM and (c) Li|SPE|NCM cell at 50 °C.

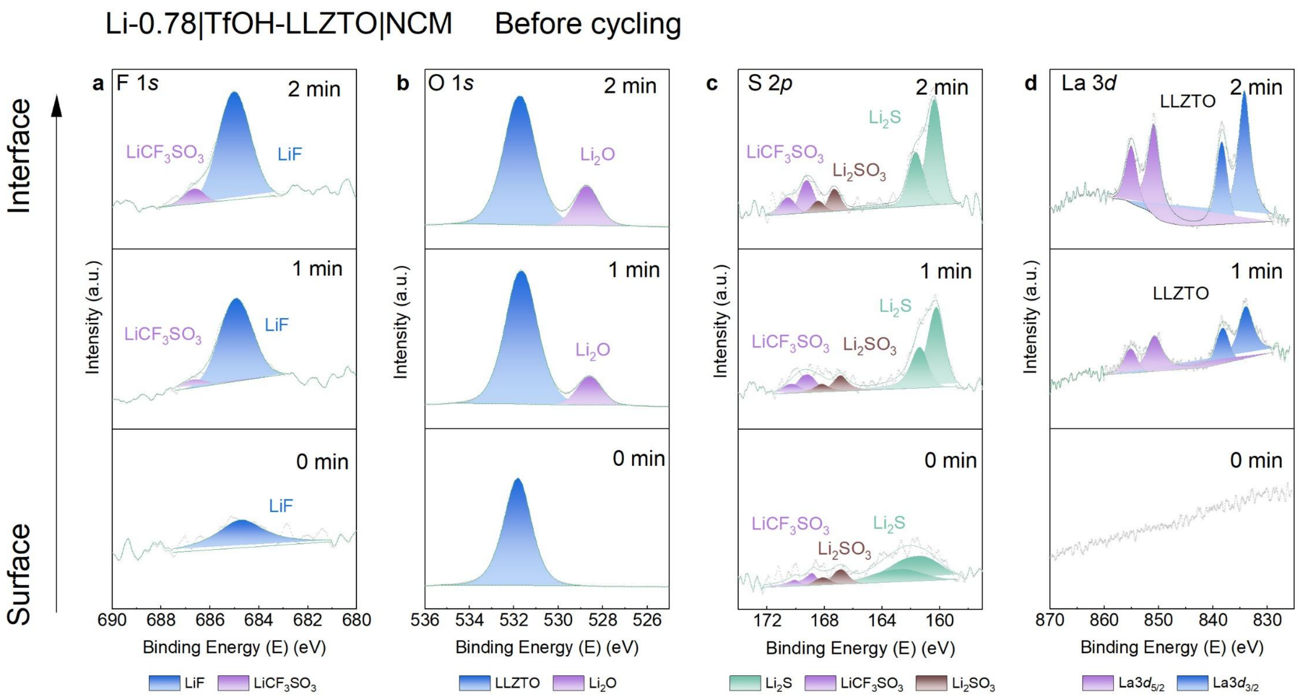

Figure S20. XPS spectra of (a) F 1*s*, (b) O 1*s*, (c) S 2*p* and (d) La 3*d* of Li-0.78 on TfOH-LLZTO with various etching time of 0, 1, and 2 min before Li-0.78|TfOH-LLZTO|NCM cycling.

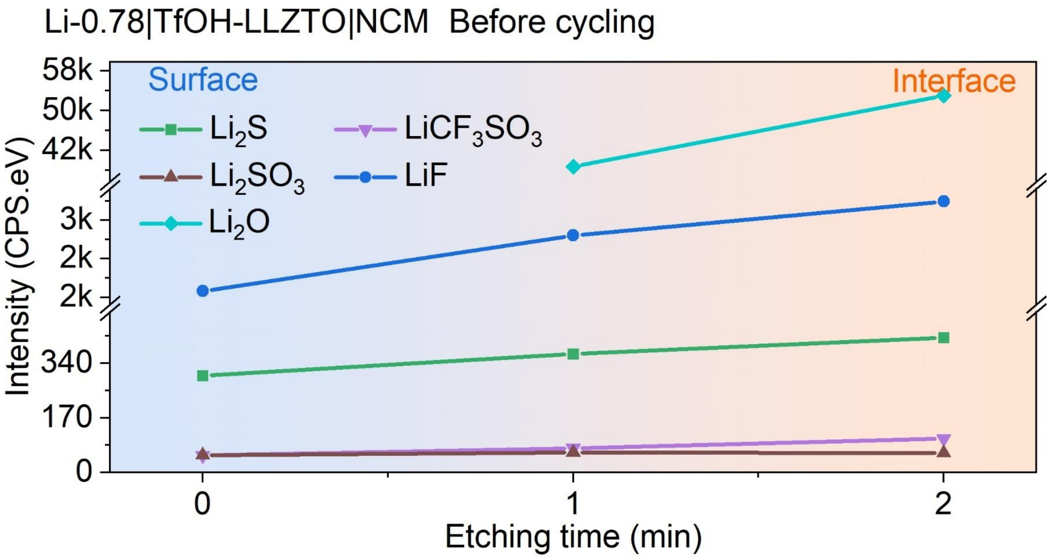

Figure S21. The intensity of different components originating from the related peaks in the F 1*s*, O 1*s*, and S 2*p* spectra before cycling.

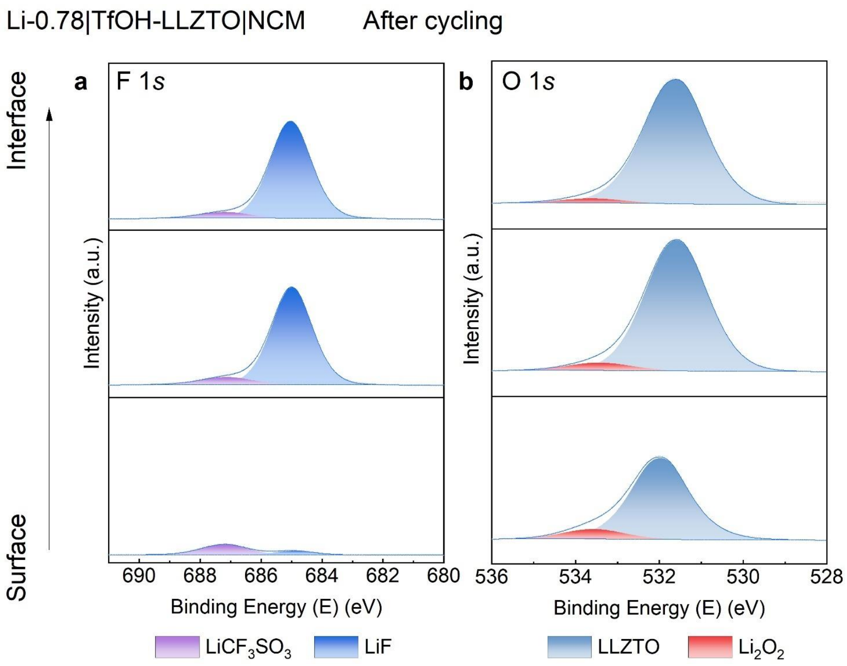

Figure S22. XPS spectra of (a) F 1*s* and (b) O 1*s* of Li-0.78 on TfOH-LLZTO after Li-0.78|TfOH-LLZTO|NCM cycling for 170 cycles under 0.14 mA cm^{-2} at 25 °C.

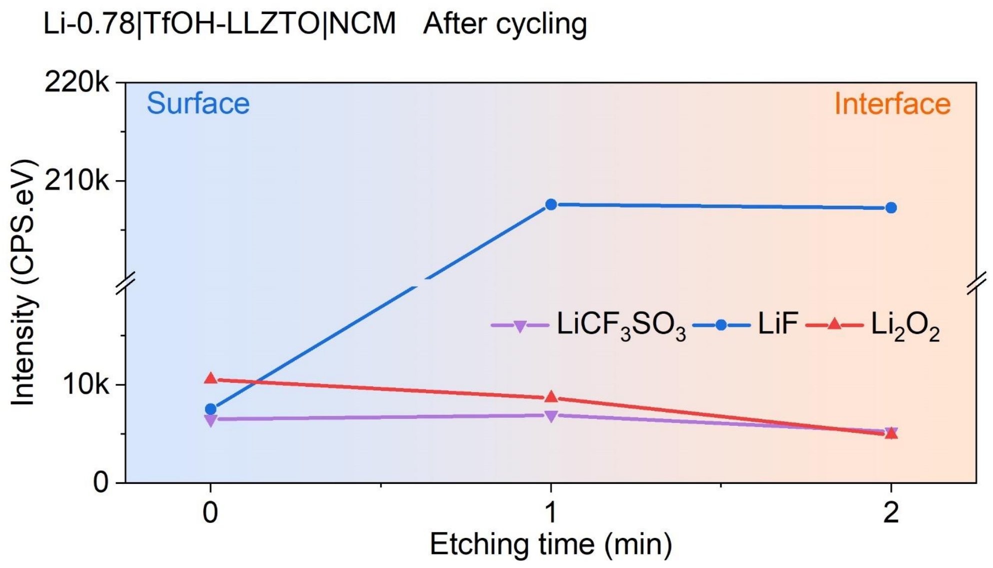

Figure S23. The intensity of different components originating from the related peaks in the F 1*s* and O 1*s* spectra after cycling for 170 cycles under 0.14 mA cm^{-2} at 25 °C.

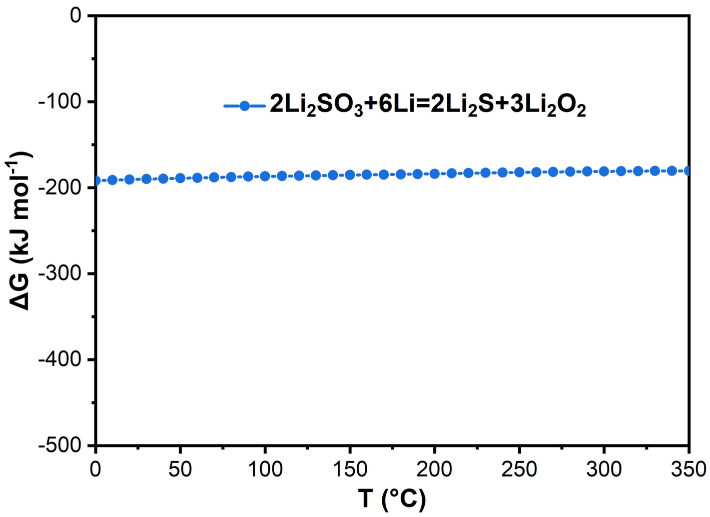

Figure S24. Changes in Gibbs free energy of the reaction Equation S4.

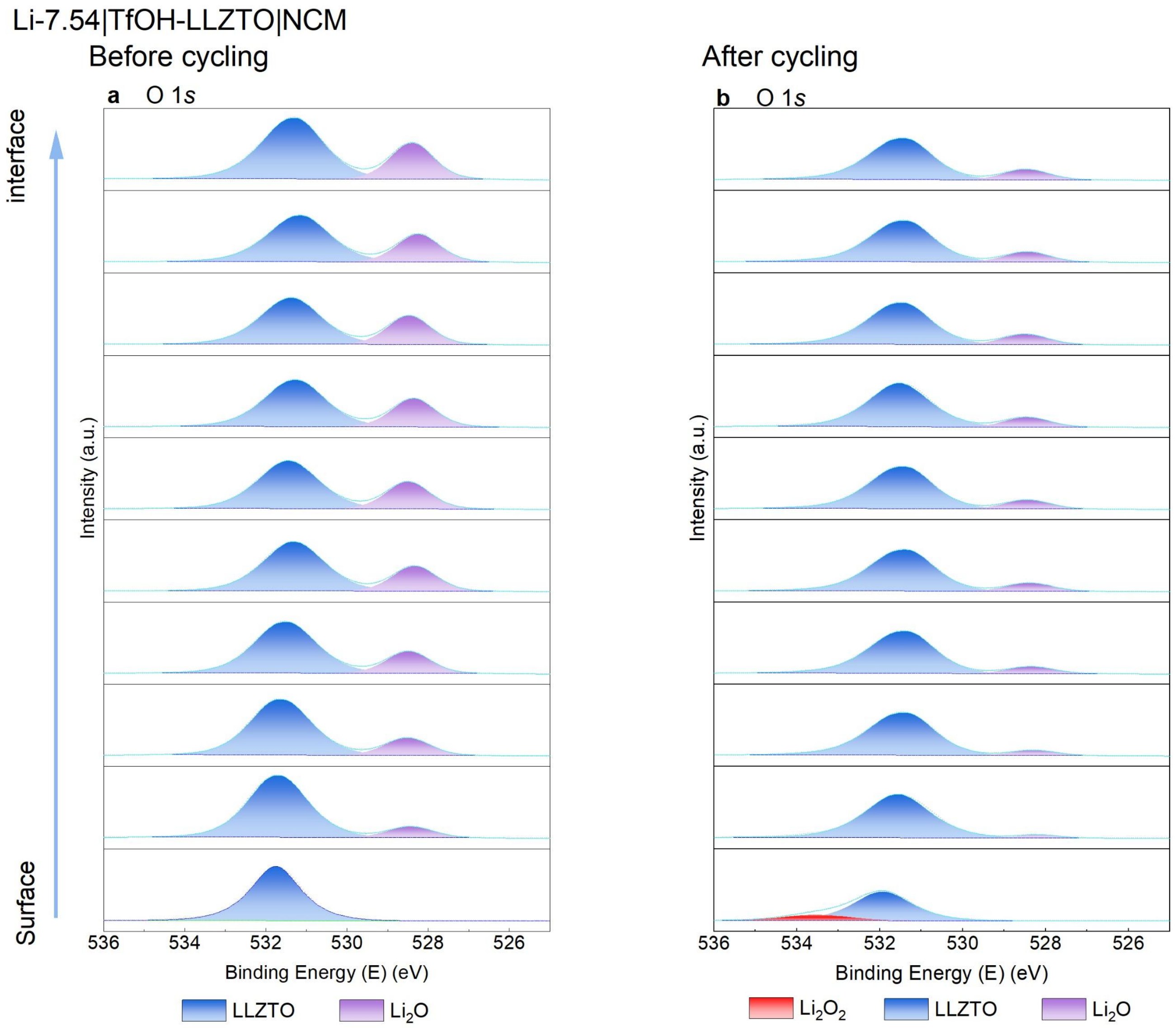

Figure S25. XPS spectra of O 1*s* of Li-7.54 on TfOH-LLZTO (a) before and (b) after Li-7.54|TfOH-LLZTO|NCM cycling for 170 cycles under 0.14 mA cm^{-2} at 25 °C.

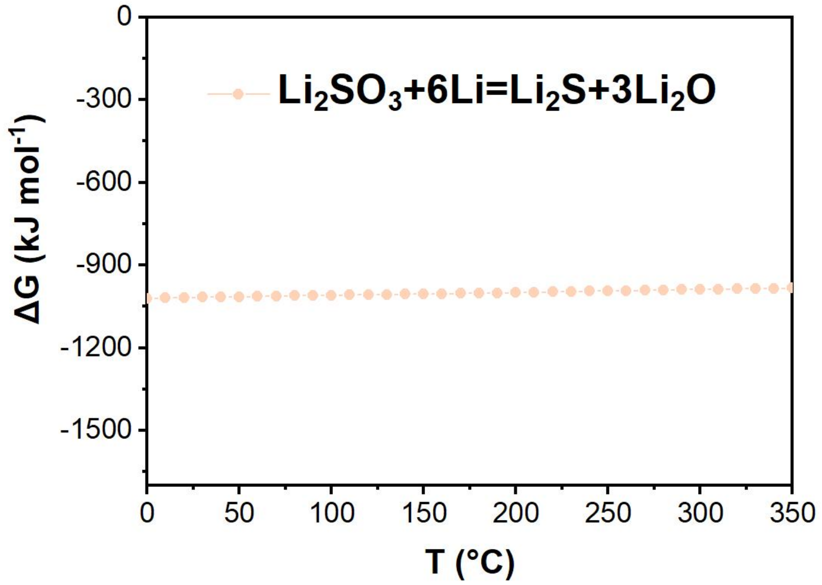

Figure S26. Changes in Gibbs free energy of the reaction Equation S5.

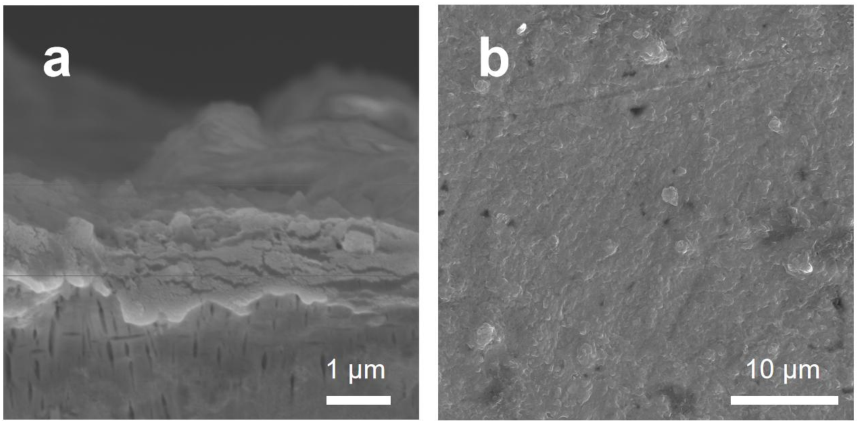

Figure S27. (a) Cross-view SEM and (b) Top-view SEM images of interfaces composed of LLZTO and lithium metals of 0.78 μm after cycling for 170 cycles under 0.14 mA cm^{-2} at 25 °C.

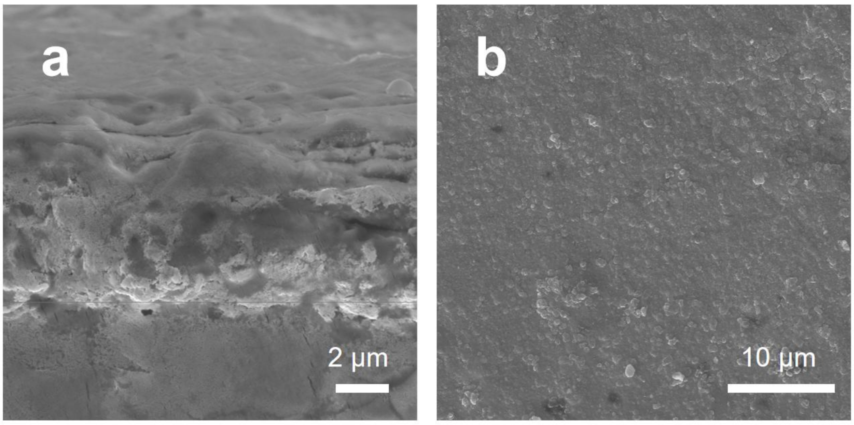

Figure S28. (a) Cross-view SEM and (b) Top-view SEM images of interfaces composed of LLZTO and lithium metals of 7.54 μm after cycling for 170 cycles under 0.14 mA cm^{-2} at 25 °C.

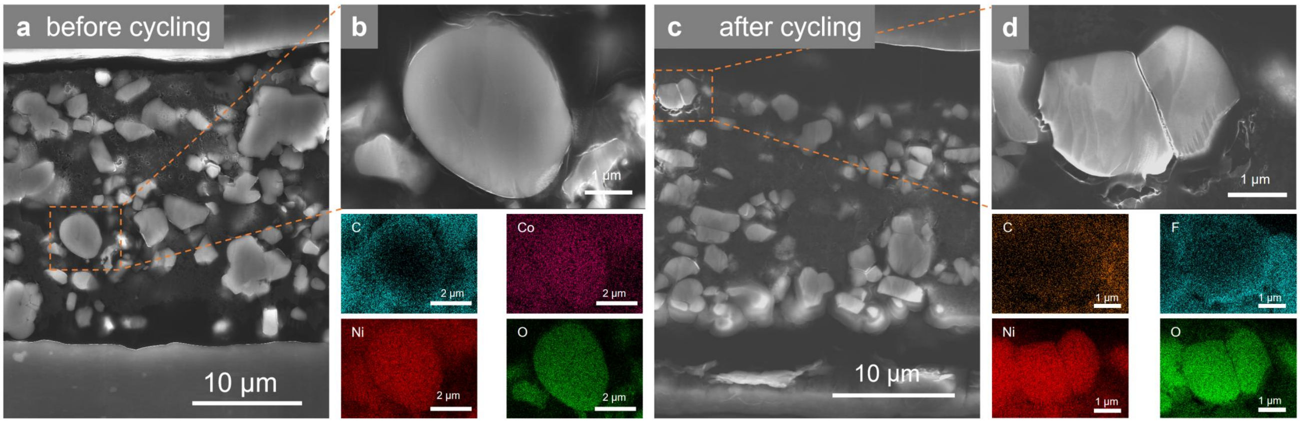

Figure S29. Cross-view FIB-SEM and EDS images of the NCM electrode (a, b) before cycling and (c, d) after cycling for 10 cycles under 0.25 mA cm^{-2} at 25 °C.

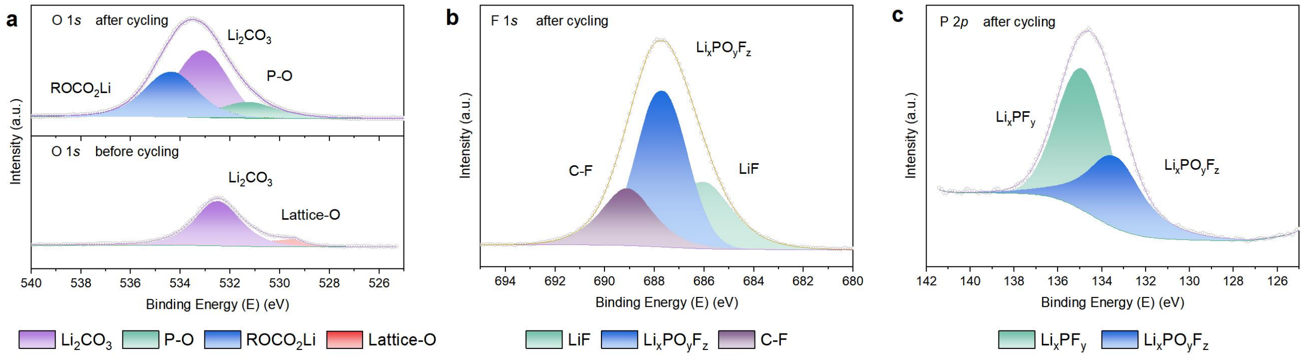

Figure S30. XPS spectra of the NCM before and after cycling. (a) XPS spectra of O 1*s* element for the NCM before and after cycling. XPS spectra of (b) F 1*s* and (c) P 2*p* elements for the NCM after cycling for 10 cycles under 0.25 mA cm^{-2} at 25 °C.

II. Supplementary Notes

Note S1.

Some potential reactions between TfOH and air-LLZTO.[22]

$2HCF_3SO_3+Li_2CO_3=2LiCF_3SO_3+CO_2+H_2O$ (Equation S1)

$LiCF_3SO_3 \rightarrow LiF+C\text{-}SO_x$+others (Equation S2)

Note S2.

Some potential reactions between Li and TfOH-LLZTO.

$2LiCF_3SO_3+16Li=3Li_2O+6LiF+Li_2S+Li_2SO_3+Li_2C_2$ ΔG=-13.5 kJ mol^{-1} T=300 °C (Equation S3)

The Gibbs free energy of the Equation S3 at 300 °C calculated by Gaussian is -319 eV, indicating that the equation is reasonable.

$2Li_2SO_3+6Li=2Li_2S+3Li_2O_2$ (Equation S4)

$Li_2SO_3+6Li=Li_2S+3Li_2O$ (Equation S5)

The Gibbs free energy of Equation S4-5 calculated using HSC 6.0 is less than 0 (0-300 °C), indicating that these equations are reasonable (as shown in Figure S24, 26).

III. Supplementary Tables

Table S1. The proportion of elements in the line scan mode of EDS in **Figure 1b**.

Element	Weight Percent/wt%	Atomic percentage/at%	Net Intensities
C	12.39	37.21	65.56
O	18.88	42.56	203.82
F	0.56	1.06	6.44
Ta	10.56	2.11	88.71
Zr	8.75	3.46	70.03
S	1.07	1.21	16.23
La	47.78	12.4	158.76

Table S2. Literature review of solid-state lithium-metal batteries based on solid-state electrolytes.

Battery structure	positive electrode	N/P ratio	Working temperat-ure [°C]	Current-rates [C]	Current density [mA cm^{-2}]	Active material loading [mg cm^{-2}]	Voltage [V]	Specific capacity [mAh g^{-1}]	Cycling number	Reten-tion [%]	Refs.
LLZTO@C	LFP	Unlimited Li	40	0.2	0.051	1.5	2.8-3.8	154.4	150	89	[5]
Li-Sr-1%	NCM811	Unlimited Li	25	0.2	0.15	4.17	2.7-4.3	192	100	82	[6]
LLZT-Ta	LFP	Unlimited Li	25	0.2	0.14	4	2.4-4	153	100	-	[8]
	NCM0.83	Unlimited Li	25	0.2	0.144	4	3-4.3	193	150	81.8	
LLZT-Al	LFP	Unlimited Li	25	0.1	0.08	4.8	2.4-4	-	500	-	[7]
	NCM0.83	Unlimited Li	25	0.2	0.17	4.8	3-4.3	200	100	90.2	
PPA-LLZTO	LFP	Unlimited Li	60	1	0.255	1.5	2.2-4.2	149.3	500	92.3	[9]
$Li\|InCl_3$	LFP	Unlimited Li	25	0.5	0.16	2	2.8-3.8	150.9	475	97.8	[10]
M-Li	LFP	Unlimited Li	25	0.5	0.255	3	2.7-3.9	-	100		[11]
Li-Mxene	LFP	Unlimited Li	25	0.5	0.208	2.45	2.5-4.2	148	100	94	[12]
LPF-LLZTO	NCM811	Unlimited Li	30	0.5	0.27	3	3-4.2	175	400	82	[13]
LLZTO-LBF	LFP	Unlimited Li	60	0.394	0.4	5.8	2.5-4	110	320	-	[14]
	LFP	Unlimited Li	60	0.986	1	5.8	-	-	200	-	
	NCM523	Unlimited Li	25	0.12	0.2	3	2.8-4.2	-	120	-	
ITO@LGLZO	LFP	Unlimited Li	25	0.2	0.102	3	2.5-4.0	150	200	90	[15]
LNO10	NCM811	Unlimited Li	25	0.2	0.072	2	2.7-4.3	178.8	115	80	[16]
	LFP		25	1	0.34	2	2.5-4.0	148.5	500	80	
Li-Na	LFP	Unlimited Li	60	-	0.1	1.5	2.0-3.8	148.3	150	71.1	[17]
			60	-	0.2	1.5	2.0-3.8	146	200	-	
Ga	LFP	Unlimited Li	60	-	0.15	2	2.4-3.8	130	440	-	[18]
Li-FEC	LFP	Unlimited Li	25	0.2	0.119	3.5	-	152.7	200	90.3	[19]
LPO@LLZTO	LFP	Unlimited Li	25	1.5	0.1	1	2.8-4.1	-	400	88	[20]

NaH_2PO_2-LLZTO	NCM811	Unlimited Li	25	0.2	0.2	5.6	3.0-4.5	155	-	-	[3]
		Unlimited Li	25	0.5	0.108	1.2	3.0-4.3	185.8	500	62	
		Unlimited Li	25	0.5	0.108	1.2	3.0-4.5	193	300	79	
		Unlimited Li	25	0.2	0.108	3	3.0-4.5	176.6	120	69.5	
		4	25	0.2	0.108	3	3.0-4.5	162.8	100	81.2	
LLZO/PCE	LFP	Unlimited Li	25	0.1	0.047	2.61	2.5-4.0	164	300	65	[4]
	NCM111	1.2	25	0.1	0.034	2.00	2.8-4.5	167	26	-	
	LFP	5.9	25	0.1	0.034	2.01	2.5-4.0	165	186	78	
	LFP	5.3	25	0.1	0.036	2.13	2.5-4.0	162	172	-	
	LFP	2.7	25	0.1	0.038	2.22	2.5-4.0	164	67	-	
	LFP	1.1	25	0.1	0.038	2.26	2.5-4.0	163	32	-	
	LFP	1.4	25	0.05	0.041	4.88	2.5-4.0	171	10	-	
VLSA CSE	NCM523	1.18	60	0.2	0.414	11.5	2.7-4.3	164.3	158	80	[2]
**TfOH-LLZTO**	**LFP**	**28.6**	**25**	**2**	**1.05**	**3.1**	**2.5-4.0**	**120.8**	**500**	**81**	**This work**
	**NCM811**	**0.3**	**25**	**0.2**	**0.28**	**7.8**	**2.7-4.3**	**188.3**	**70**	**55.0**	
	**NCM811**	**0.5**	**25**	**0.2**	**0.28**	**7.8**	**2.7-4.3**	**189.0**	**170**	**59.3**	
	**NCM811**	**1.0**	**25**	**0.2**	**0.28**	**7.8**	**2.7-4.3**	**192.5**	**170**	**76.7**	
	**NCM811**	**3.4**	**25**	**0.2**	**0.28**	**7.8**	**2.7-4.3**	**190.9**	**170**	**76.6**	
	**NCM811**	**1.1**	**25**	**2**	**2.35**	**6.448**	**2.7-4.3**	**165.5**	**500**	**74.3**	

Supplementary References

1. Zou P, Wang C, He Y, Xin HL. Broadening solid ionic conductor selection for sustainable and earth-abundant solid-state lithium metal batteries. *Energy Environ Sci* **16**, 5871-5880 (2023).
2. Wang J, *et al.* Highly conductive thin composite solid electrolyte with vertical $Li_7La_3Zr_2O_{12}$ sheet arrays for high-energy-density all-solid-state lithium battery. *Chem Eng J* **450**, 137994-138002 (2022).
3. Liu Y, *et al.* Enable high reversibility of Fe/Cu based fluoride conversion batteries via interfacial gas release and detergency of garnet electrolytes. *Mater Today* **61**, 65-77 (2022).
4. Chen S, *et al.* All-solid-state batteries with a limited lithium metal anode at room temperature using a garnet-based electrolyte. *Adv Mater* **33**, 2002325-2002335 (2021).
5. Chen L, *et al.* Excellent Li/Garnet Interface Wettability Achieved by Porous Hard Carbon Layer for Solid State Li Metal Battery. *Small* **18**, 2106142-2106152 (2022).
6. He X, *et al.* Tuning Interface Lithiophobicity for Lithium Metal Solid-State Batteries. *ACS Energy Lett* **7**, 131-139 (2021).
7. Guo S, *et al.* Coordination-assisted precise construction of metal oxide nanofilms for high-performance solid-state batteries. *J Am Chem Soc* **144**, 2179-2188 (2022).
8. Guo S, *et al.* Interface engineering of a ceramic electrolyte by Ta_2O_5 nanofilms for ultrastable lithium metal batteries. *Adv Funct Mater* **32**, 2201498-2201508 (2022).
9. Guo C, *et al.* Grafting of Lithiophilic and Electron-Blocking Interlayer for Garnet-Based Solid-State Li Metal Batteries via One-Step Anhydrous Poly-Phosphoric Acid Post-Treatment. *Adv Funct Mater* **33**, 2213443-2213453 (2022).
10. Leng J, *et al.* A facile and low-cost wet-chemistry artificial interface engineering for garnet-based solid-state Li metal batteries. *Nano Energy* **101**, 107603-107613 (2022).
11. Wu W, *et al.* Magnetic actuation enables programmable lithium metal engineering. *Adv Energy Mater* **12**, 2200999-2201009 (2022).
12. Wen J, *et al.* A Lithium-MXene Composite Anode with High Specific Capacity and Low Interfacial Resistance for Solid-State Batteries. *Energy Storage Mater* **45**, 934-940 (2021).
13. Yang X, *et al.* From Contaminated to Highly Lithiated Interfaces: A Versatile Modification Strategy for Garnet Solid Electrolytes. *Adv Funct Mater* **33**, 2209120-2209128 (2022).
14. Cai M, Jin J, Xiu T, Song Z, Badding ME, Wen Z. In-situ constructed lithium-salt lithiophilic layer inducing bi-functional interphase for stable LLZO/Li interface. *Energy Storage Mater* **47**, 61-69 (2022).
15. Gao M, *et al.* Constructing a Multifunctional Interlayer toward Ultra-High Critical Current Density for Garnet-Based Solid-State Lithium Batteries. *Adv Funct Mater* **33**, 2300319-2300327 (2023).
16. Xu C, *et al.* Built-in superionic conductive phases enabling dendrite-free, long lifespan and high specific capacity of composite lithium for stable solid-state lithium batteries. *Energy Environ Sci* **16**, 1049-1061 (2023).
17. Zhang Y, Meng J, Chen K, Wu H, Hu J, Li C. Garnet-Based Solid-State Lithium Fluoride Conversion Batteries Benefiting from Eutectic Interlayer of Superior Wettability. *ACS Energy Lett* **5**, 1167-1176 (2020).
18. Meng J, Zhang Y, Zhou X, Lei M, Li C. Li_2CO_3-affiliative mechanism for air-accessible interface engineering of garnet electrolyte via facile liquid metal painting. *Nat Commun* **11**, 3716-3728 (2020).
19. Jiang T, He P, Wang G, Shen Y, Nan C-W, Fan L-Z. Solvent-Free Synthesis of Thin, Flexible, Nonflammable Garnet-Based Composite Solid Electrolyte for All-Solid-State Lithium Batteries. *Adv Energy Mater* **10**, 1903376-1903386 (2020).
20. Deng T, *et al.* Tuning the anode-electrolyte interface chemistry for garnet-based solid-state Li metal batteries. *Adv Mater* **32**, 2000030-2000040 (2020).

21. He F, Tang W, Zhang X, Deng L, Luo J. High Energy Density Solid State Lithium Metal Batteries Enabled by Sub-5 microm Solid Polymer Electrolytes. *Adv Mater* **33**, 2105329-2105338 (2021).
22. Yu W, Yu Z, Cui Y, Bao Z. Degradation and Speciation of Li Salts during XPS Analysis for Battery Research. *ACS Energy Lett* **7**, 3270-3275 (2022).
