# Supplementary material for: Interface engineering enabling thin lithium metal electrodes down to 0.78 μm for garnet-type solid-state batteries
Source: Nat Commun. 2024 Nov 15;15:9920. doi: 10.1038/s41467-024-54234-w (PMC11568204; doi:10.1038/s41467-024-54234-w)
Supplement: Supplementary file 3 — Description of Additional Supplementary Files [file 41467_2024_54234_MOESM3_ESM.pdf]

Description of Additional Supplementary Files

Supplementary Data 1: The model data of Li|Li_2CO_3 interface calculated by density functional theory.

Supplementary Data 2: The model data of Li|$Li_7La_3Zr_2O_{12}$ interface calculated by density functional theory.

Supplementary Data 3: The model data of Li|LiF interface calculated by density functional theory.

Supplementary Data 4: The model data of Li|$LiCF_3SO_3$ interface calculated by density functional theory.
